# Omeprazole and adenocarcinoma in the stomach of rats submitted to duodenogastric reflux. Is there a protective effect? ^1^

**DOI:** 10.1590/s0102-865020200090000004

**Published:** 2020-09-30

**Authors:** Rosângela Lucinda Rocha Monteiro, Maria Aparecida Marchesan Rodrigues Kobayasi, Marina Rachel de Araujo, Dafny Rocha Monteiro, Nelson Adami Andreollo

**Affiliations:** I Master, Assistant Professor, School of Medicine, Universidade do Vale do Sapucaí (UNIVÁS), Pouso Alegre-MG, Brazil. Conception and design of the study, technical procedures, acquisition and interpretation of data, statistics analysis, manuscript preparation.; II PhD, Full Professor, Department of Pathology, Medical School, Universidade Estadual Paulista (UNESP), Botucatu-SP, Brazil. Analysis and interpretation of data, histopathological examinations, statistics analysis, manuscript preparation, critical revision.; III Biologist, Experimental Surgery Laboratory, School of Medical Sciences, Universidade Estadual de Campinas (UNICAMP), Brazil. Acquisition of data, technical procedures.; IV Graduate student, Centro Universitário de Faculdades de Ensino Associadas (UNIFAE), Sao Joao da Boa Vista-SP, Brazil. Acquisition of data, technical procedures.; V PhD, Full Professor, Digestive Diseases Surgical Unit and Gastrocentro, School of Medical Sciences, UNICAMP, Campinas-SP, Brazil. Conception and design of the study, interpretation of data, manuscript preparation, critical revision.

**Keywords:** Duodenogastric Reflux, Carcinogenesis, Nitrites, Omeprazole, Rats

## Abstract

**Purpose:**

To investigate the role of omeprazole and nitrites on the gastric mucosa of rats submitted to specific techniques to induce duodenogastric reflux.

**Methods:**

One hundred and twenty Wistar rats were divided into three groups: Group I (n=40) -gastrotomy; Group II (n=40) - duodenogastric reflux after gastrojejunoanastomosis latero-lateral (DGR); Group III (n=40) - retrograde duodenogastric reflux through the pylorus (DGR-P). The groups were divided into 4 subgroups of 10 animals, respectively treated for 16 weeks with water, omeprazole 1.6 mg / rat / day, nitrite 600 mg / kg / day and omeprazole plus nitrite simultaneously.

**Results:**

The proliferative lesions found were: squamous hyperplasia - 69.1%, adenomatous hyperplasia in the anastomosis - 29.1% and prepyloric adenomatous hyperplasia - 42.5%. Adenocarcinomas were registered in 7 animals (5.8%): one in Group I (omeprazole plus nitrite), two in Group II (omeprazole and nitrite plus omeprazole) and four in Group III (water, nitrite, omeprazole and omeprazole plus nitrite).

**Conclusions:**

The occurrence of squamous hyperplasia, adenomatous hyperplasia and adenocarcinoma increased after gastrojejunal anastomoses, which cause duodenogastric reflux. The association of omeprazole did not protect the development of proliferative lesions and cancer induced by duodenogastric reflux in rats.

## Introduction

Omeprazole was introduced for clinical use in 1989; it is a potent proton pump inhibitor (PPI), which, by decreasing gastric secretion and changing the activity of H + / K + - ATP, reduces daily acid production by 95%. Omeprazole and other proton pump inhibitors (PPI's), such as lanzoprazole, pantoprazole, rabeprazole, esomeprazole and more recently dexlanzoprazole, are widely used in the treatment of esophagitis, gastritis, prophylaxis of stress ulcers, stomach protection in chronic use of non-steroidal anti-inflammatory drugs (NSAIDs), bleeding from the upper digestive tract, Zollinger-Ellison syndrome and peptic ulcers ^1^ . Recently, some local effects of prolonged use of PPI's have been recorded, such as atrophic gastritis, chronic *Helicobacter pylori* infection, hypergastrinemia, and development of gastric polyps resulting from prolonged acid suppression, which have led to concerns about the increased risk for gastric cancer ^2^ ^-^ ^4^ .

Studies in rodents have shown that potent inhibition of gastric acid secretions induces gastric cancer, associated with secondary hypergastrinemia and resulting in enterochromaffin-like cell hyperplasia ^5^ ^,^ ^6^ . In addition, the blockage of acid secretion causing chronic hypochlorhydria and hypergastrinemia, results in the proliferation of gastric mucosa, chronic inflammation, decreased gastric glandular tissue, gastric atrophy and the appearance of intestinal metaplasia, associated with chronic infection by *Helicobacter pylor* , ^3^ ^,^ ^7^ ^,^ ^8^ ^,^ ^12^ .

The gastric mucosa is particularly susceptible to the development of proliferative lesions when subjected to reflux of the duodenal contents, and some studies are directed to assess the possible relationship between primary duodenogastric reflux and the occurrence of neoplasia ^9^ ^,^ ^10^ . Furthermore, clinical and experimental studies have been carried out to investigate whether surgical techniques that promote duodenogastric reflux may be involved in the development of gastric cancer ^11^ ^,^ ^12^ . The intensity of glandular atrophy and intestinal metaplasia is directly related to the reflux of duodenal secretions to the gastric mucosa ^13^ ^,^ ^14^ .

Nitrites and nitrates are inorganic substances found in nature, in a wide variety of food products consumed by man, in drinking water and fertilizers. They are widely used in the food industry as preservatives for meat, canned and smoked products. They are the precursors of nitrosamines and n-nitrous compounds, substances, considered as carcinogenic to humans and laboratory animals ^15^ ^-^ ^17^ .

The aim of this study was to investigate the effects of omeprazole and nitrites on the gastric mucosa of rats submitted to duodenogastric reflux.

## Methods

The research project was approved by the Animal Research Ethics Committee, Universidade Estadual de Campinas (UNICAMP).

The study included 120 male Wistar rats, with approximately 8 weeks and weight between 180 and 280 grams. The induction of gastric duodenal reflux was obtained through a surgical procedure, as previously described ^9^ ^,^ ^10^ ^,^ ^18^ .

**Figure 1:**
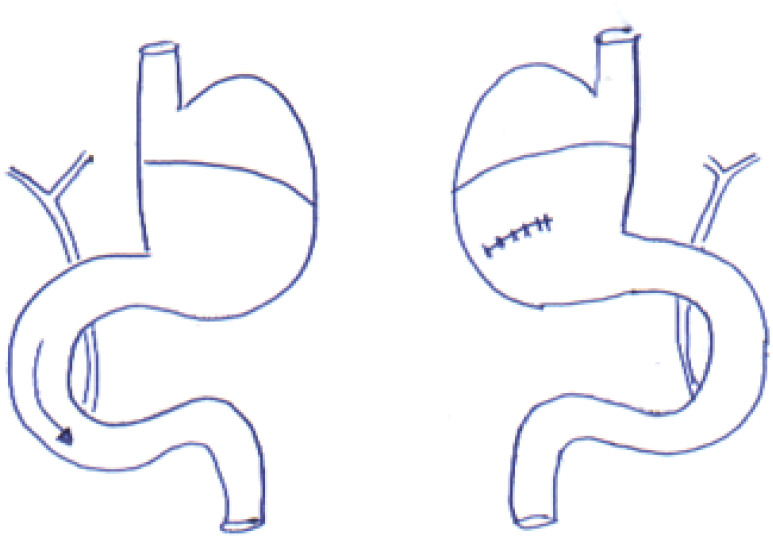
Group I (Control).

Three groups of animals were studied:

**Group I (control C)** - Forty animals were submitted to 1 cm gastrotomy on the posterior wall of the glandular stomach transversally the gastric axis; manipulation of the intestinal loops was performed, followed by continuous suture in a single plane with wire 6-0 polypropylene ( Fig. 1 ).

**Figure 2:**
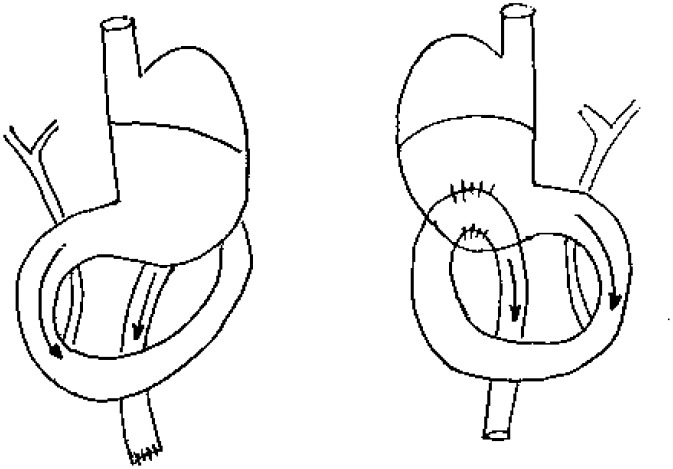
Group II (DGR).

**Group II (DGR)** - Forty animals were submitted to induction of gastric duodenal reflux by gastrojejunoanastomosis of 1 cm in extension, 4 cm away from the Treitz angle, in isoperistatic direction of the loop, and in the posterior wall of the glandular stomach ( Fig. 2 ).

**Figure 3:**
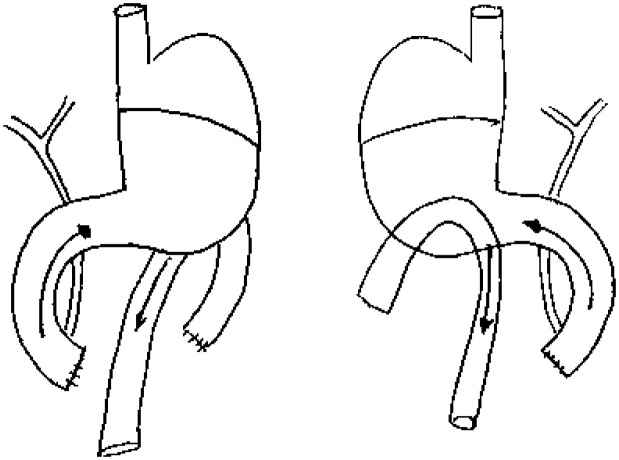
Group III (DGR-P).

**Group III (DGR-P)** - Forty animals were submitted to gastrojejunoanastomosis similar to group II, but with section and ligation of the afferent loop, inducing retrograde duodenogastric reflux by the pylorus ( Fig. 3 ) ^9^ ^,^ ^10^ ^,^ ^18^ . After the procedures, the animals ingested only water during the initial 24 hours after surgery and free access to the feed after this period.

Omeprazole, nitrite or a combination of both started after 20 days postoperatively, according to the subgroup to which they belonged. The three groups were subdivided into 4 subgroups of 10 animals each, according to the drug administered together with water and feed.

Thus, in each of the 3 groups, the control subgroup (C) was exposed to water only, the omeprazole subgroup (O) received 1.6 mg / rat / day of omeprazole, the nitrite subgroup (N) received 600 mg / kg / nitrite day and the omeprazole plus nitrite subgroup (NO) received both substances in the water and offered in the same dosage as above. The animals were weighed weekly, during the observation period and euthanized after 16 weeks, with the same anesthetic procedure. Obtaining the surgical specimen and analyzing the macroscopic changes found followed a previous research protocol, described by Monteiro *et al* . ^18^ .

The protocol for the histological analysis was prepared, including the following criteria ^11^ ^,^ ^15^ ^,^ ^18^: 

**Squamous Hyperplasia (SH)** - Squamous epithelium thicker than normal twice or more, with hyperkeratosis.

**Adenomatous Hyperplasia (AH)** – Proliferation of glandular structures without cellular atypia, with endophytic or exophytic growth to the gastric wall.

**Adenocarcinoma (AC)** - Proliferation of glandular structures with structural disorganization, cellular atypia and invasive growth, endophytic or exophytic to the gastric wall.

The statistical analysis employed results of frequencies and percentages. Groups and treatments were compared using Logistic Regression and Fischer Exact Test. The level of significance adopted was 5%.

## Results

The macroscopic alterations found were identified as polypoid lesions or sessile, of varied sizes, at the gastrojejunal anastomosis and some in the prepyloric mucosa in group III. No macroscopic lesions were identified in control group (Group I), except for discrete prominence of the mucosa at the gastrotomy location. The proliferative lesions found in the Group I (control) are shown in Table 1: 

**Table 1:** Frequency of microscopic lesions (%) and respective locations in surgical specimens in Group I, represented by Squamous Hyperplasia (SH), Adenomatous Hyperplasia - stoma and prepyloric (AH) and Adenocarcinoma (AC), in animals ingesting water, nitrite, omeprazole, and omeprazole plus nitrite.

Group I (C)	N	Squamous stomach SH (%)	Stoma AH (%)	Stoma AC (%)	Prepyloric AH (%)
Water	10	0(0%)	0(0%)	0(0%)	0(0%)
Nitrite	10	9(90%)	2(20%)	0(0%)	4(40%)
Omeprazole	10	6(60%)	3(30%)	0(0%)	4(40%)
Omeprazol plus Nitrite	10	8(80%)	1(10%)	1(10%)	5(50%)
**Total**	**40**	**23 (57.5%)**	**6 (15%)**	**1 (2.5%)**	**14 (35%)**

The proliferative lesions found in Group II (DGR), are shown in Table 2: 

**Table 2:** Percentages of microscopic lesions (%) and respective locations in surgical pieces in the Group II, represented by Squamous Hyperplasia (SH), Adenomatous Hyperplasia - stoma and prepyloric (AH) and Adenocarcinoma (AC), in animals ingesting water, nitrite, omeprazole, and omeprazole plus nitrite.

Group II (DGR)	N	Squamous stomach HE (%)	Stoma AH (%)	Stoma AC (%)	Prepyloric AH (%)
Water	10	0(0%)	4(40%)	0(0%)	5(50%)
Nitrite	10	8(80%)	3(30%)	0(0%)	5(50%)
Omeprazole	10	10(100%)	6(60%)	1(10%)	6(60%)
Omeprazol plus Nitrite	10	10(100%)	1(10%)	1(10%)	3(30%)
**Total**	**40**	**28 (70%)**	**14 (35%)**	**2 (5%)**	**19 (47.5%)**

The proliferative lesions found in group III (DGR + P) are shown in Table 3: 

**Table 3:** Percentages of microscopic lesions (%) and respective locations in surgical pieces in the Group III, represented by Squamous Hyperplasia (SH), Adenomatous Hyperplasia - stoma and prepyloric (AH) and Adenocarcinoma (AC), in animals ingesting water, nitrite, omeprazole, and omeprazole plus nitrite.

Group III (DGR-P)	N	Squamous stomach SH (%)	Stoma AH (%)	Stoma AC (%)	Prepyloric AH (%)
Water	10	2(20%)	7(70%)	1(10%)	3(30%)
Nitrite	10	10(100%)	1(10%)	1(10%)	3(30%)
Omeprazole	10	10(100%)	6(60%)	1(10%)	8(80%)
Omeprazol plus Nitrite	10	10(100%)	1(10%)	1(10%)	5(50%)
**Total**	**40**	**32 (80%)**	**15 (37.5%)**	**4 (12.5%)**	**19 (47.5%)**

**Figure 4:**
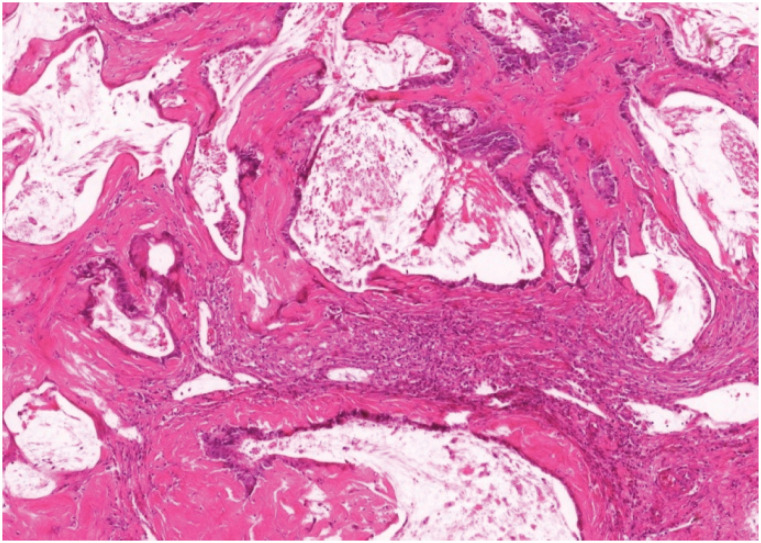
Mucinous adenocarcinoma at the gastrojejunal mucosa (HE, x400).

Adenocarcinomas were recorded in seven animals (5.8%). The macroscopic lesions had a vegetating aspect on the mucosa of the gastrojejunal anastomosis (stoma), and the histological examination showed cellular atypia and cystic lesions, being classified as mucinous adenocarcinoma ( Fig. 4 ): 1 animal in Group I (omeprazole plus nitrite subgroup), 2 animals in Group II (omeprazole and omeprazole plus nitrite subgroups) and four animals in Group III (water, nitrite, omeprazole and omeprazole plus nitrite subgroups) ( Tables 4 and 5 ).

**Table 4:** Percentagens of mucinous adenocarcinoma (%) in the anastomosis (stoma): Group I (Gastrotomy), Group II (Gastric duodenal reflux) and Group III (Gastric duodenal reflux through the pylorus) with water, nitrite, omeprazole and omeprazole plus nitrite.

Groups	I	II	III
Water	0(0%)	0(0%)	1(0.83%)
Nitrite	0(0%)	0(0%)	1(0.83%)
Omeprazole	0(0%)	1(0.83%)	1(0.83%)
Omeprazol plus Nitrite	1(0.83%)	1(0.83%)	1(0.83%)
Total	1(0.83%)	2(1.6%)	4(3.3%)

**Table 5:** Percentages of microscopic lesions in Groups I, II and III. Anastomosis (A); Prepyloric area (Pre).

Groups	I	II	III	Total (%)
Squamous hyperplasia	23	28	32	83(69.1%)
Adenomatous hyperplasia (A)	6	14	15	35(29.1%)
Adenomatous hyperplasia (Pre)	13	19	19	51(42.5%)
Adenocarcinoma	1	2	4	7(5.8%)

Therefore, 5.8% of adenocarcinomas were recorded. The use of isolated nitrite provoked the occurrence of adenocarcinoma in only 1 animal (0.83%) in group III. Furthermore, in animals that received omeprazole, adenocarcinomas were also registered in groups II and III, with partial and total reflux, respectively. However, the presence of adenocarcinoma was registered in one animal (0.83%) from each group, which received the association omeprazol plus nitrite. The statistical analysis employing the Fischer test comparing the occurrence of adenocarcinomas and the treatments employed, showed p = 0.8340 - p> 0.05, therefore without statistically significant difference.

The Table 5 below demonstrates the percentages of histological changes (%) in the three groups studied:

The statistical analysis comparing the occurrence of squamous hyperplasia between the groups (logistic regression) showed a statistically significant difference between group III and group I (p=0.0121 - p<0.05). Comparing the other groups, there was no statistically significant difference (group II *vs* . group I - p=0.1628 - p>0.05, group II *vs* . group II - p=0.1024).

However, comparing the treatments used in the three groups, there was a statistically significant difference between nitrite *vs* . water (p<0.0001 - p<0.05), omeprazole vs. water (p<0.0001 - p<0.05) and omeprazol plus nitrite. In the other comparisons (omeprazole *vs* . nitrite, omeprazol plus nitrite *vs* . nitrite and omeprazol plus nitrite *vs* . omeprazole), p>0.05.

The adenomatous hyperplasia in the anastomosis (stoma), the statistical analysis between the groups (logistic regression) showed a statistically significant difference between group III and group I (p=0.0049–p<0.05). Comparing the other groups, there was no statistically significant difference (group II *vs* . group I - p=0.0813 - p>0.05, group II *vs* . group III - p=0.0813 - p>0.05).

However, comparing the treatments used in the three groups, there were statistically significant differences between omeprazole vs. nitrite (p<0.039 - p<0.05) and omeprazol plus nitrite vs. omeprazole (p<0.0174 - p<0.05). In the other comparisons (nitrite vs. water, omeprazole vs. water, omeprazole vs. nitrite vs. water and omeprazole vs. nitrite vs. nitrite), p>0.05.

The statistical analysis of adenomatous hyperplasia in the prepyloric area between the groups (logistic regression) showed no statistical difference when comparing group II *vs* . group I (p=0.4745 - p>0.05), group III *vs* . group II (p=0.3522 - p>0.05) and group III *vs* . group I (p=0.1034 - p>0.05). However, when comparing the treatments used in the three groups, there were statistically significant differences between omeprazole *vs* . water (p=0.050 - p<0.05) and omeprazole *vs* . nitrite (p=0.0391 - p<0.05). In the other comparisons (nitrite *vs* . water, omeprazole *vs* . nitrite *vs* . water, omeprazole *vs* . nitrite *vs* . nitrite and omeprazole *vs* . nitrite *vs* . omeprazole), p>0.05.

## Discussion

The role of duodenogastric reflux in the development of benign and malignant lesions in the stomach, with or without gastric resection, has been studied for many years and has generated a large number of studies. Previous research carried out with the same protocol and published in 2006 showed that the animals in Group I (controls) did not present any type of injury. In group II, 40% of adenomatous hyperplasia lesions were observed in the anastomosis and 12% of squamous hyperplasia. In group III, 40% of adenomatous hyperplasia was obtained in the pre-pyloric mucosa, 72% of adenomatous hyperplasia in the anastomosis mucosa (stoma), 20% of squamous hyperplasia and 12% of adenocarcinoma. The final conclusions showed that provoked duodenogastric reflux induces a high frequency of proliferative lesions in the mucosa adjacent to the gastrojejunal anastomosis or in the pre-pyloric mucosa and adenocarcinoma is not a frequent event in this experimental model ^18^ .

Nitrites and nitrates are substances found widely in foods consumed by man, in drinking water and fruits and vegetables. They are often used as food additives and preservatives in processed meats, such as bacon, ham, sausages and hot dogs. The biochemical reduction of nitrates leads quickly to the formation of nitrites, and the concentration of nitrite in the stomach will be higher the more alkaline the pH. Therefore, nitrites are the active ingredient and nitrates serve as reservoirs that supply nitrites. There are numerous epidemiological studies that associate the potential risk of gastric cancer and other cancers with the intake of nitrates, nitrites and nitrosamines in the diet ^15^ ^,^ ^17^ ^,^ ^19^ ^-^ ^21^ .

Modena *et al* . ^22^ employed nitrites in an experimental model using Wistar rats, causing duodenal-esophageal-gastric reflux in the genesis of adenocarcinoma associated with Barrett's esophagus. They demonstrated that after 42 weeks of observation, in animals operated without nitrite ingestion, Barrett's esophagus was registered in 26.3% animals, while in the operated group associated with nitrite ingestion, it was found in 72.3% of the animals, and in this group, adenocarcinoma was also registered in 33.3% animals.

Moore *et al* . ^23^ used the same experimental model and compared the histopathological findings found in 20 animals that ingested only water with 19 animals that received omeprazole, being observed for six months. Among the animals that ingested water, three were recorded with Barrett's esophagus and adenocarcinoma (15%). However, in animals treated with omeprazole, three animals with Barrett's esophagus (15.7%), two animals with dysplasia (10.5%) and three other animals with adenocarcinoma in Barrett's esophagus (15.7%) were registered. The authors concluded that there was no significant difference in the development of dysplasia or adenocarcinoma among rats that received treatments with omeprazole and rats that ingested water.

In recent years, PPI's have been widely used worldwide to treat gastroduodenal diseases and omeprazole is the most consumed. In addition to the pharmacological superiority of PPI's in decreasing gastric acidity compared to other drugs, they include other advantages: in most cases they can be taken only as a single daily dose, are inexpensive, promptly available and can be purchased without a prescription, factors that lead to prolonged, inappropriate and sometimes unnecessary use, exposing individuals to adverse effects ^24^ .

In addition to the effects on the gastric mucosa, recent studies have associated the prolonged use of PPI's with serious systemic adverse effects, such as increased risk of osteoporosis-related fractures, *Clostridium difficile* infection, dementia, malabsorption of vitamins and minerals such as vitamin B12, calcium and iron, pneumonia and kidney disease ^24^ ^,^ ^25^ .

The balance between the effects of decreased gastric and esophageal inflammation with PPI's and the theoretical basis for preventing the occurrence of cancer, is unknown in animals and humans. Recent research has shown that chronic mucosal exposure to these substances is associated with intestinal metaplasia, predisposing to the development of gastric cancer, as the metaplastic epithelium tends to increase cell proliferation, and due to the deficient power of cell inactivation, that provides greater contact with mucosa of ingested carcinogenic substances. The carcinogenic mechanism would also be related to the increased production of trophic peptides, such as gastrin, in response to prolonged therapeutic hypochlorhydria, and endocrine cell hyperplasia. In the rat's stomach, this trophic action determines the appearance of carcinoid tumors and an increase in the population of enterochromafin-like cells (ECL), in addition to being related to the appearance of adenocarcinoma and squamous cell carcinoma induced by N-nitrous compounds. Nitrosamines formed by bacterial action in hypochloric stomachs have been considered an important factor in the development of gastric cancer ^22^ ^,^ ^26^ .

Other recent clinical evidence supports the association of PPI's and gastric cancer development. Retrospective case control studies from databases in western and eastern countries recently analyzed the increased risk of gastric cancer with ingestion of PPI's ^27^ ^-^ ^31^ . Cheung *et al* . ^29^ showed a positive correlation between PPI and gastric cancer in 63.000 patients with *H. pylori* who received treatment based on clarithromycin. During an average 7.6-year follow-up, 153 patients (0.24%) developed gastric cancer. The authors concluded that the use of PPI's significantly increased the risk of gastric cancer. This study is significant because it demonstrated an increased risk of gastric cancer with prolonged use of PPI's, even after successful eradication of *H. pylori* .

On the other hand, the population-based Swedish national cohort study that recruited nearly 800.000 Swedish adults using PPI's, failed to establish a causal relationship between gastric cancer and long-term use of PPI ^32^ .

Within this context, we highlight the observation by Laterza *et al* . ^33^ emphasizing that in patients in whom long-term PPI's is indicated, detailed prospective observational studies are necessary to assess the true risk of gastric cancer, identifying possible concomitant risk factors.

This study was an experimental study to evaluate the histopathological changes found in the gastric mucosa of rats submitted to duodenogastric reflux, treated with nitrites, omeprazole and omeprazole plus nitrites. Squamous hyperplasia was the most frequent change, recorded in 69.1% of the animals, followed by adenomatous hyperplasia in the prepyloric area (42.5%) and gastrojejunal anastomosis (29.1%) and adenocarcinoma (5.8%).

The occurrence of adenomatous hyperplasia in the gastrojejunal anastomosis also showed a statistically significant difference between Group III and Group I, but mainly among animals that received omeprazole and omeprazole plus nitrite. The occurrence of adenomatous hyperplasia in the prepyloric region was not statistically significant among the three groups; however, it was significant among the animals that received omeprazole. Therefore, a negative point of this study was due to the small number of animals that developed tumors, and we cannot conclude that there is a significant difference between those that received, singly, nitrites, omeprazole and omeprazole plus nitrite. Also, we cannot conclude that omeprazole and omeprazol plus nitrite increased the risk of adenocarcinoma development.

Adenocarcinoma was registered in 7 animals (5.8%), respectively in one Group I animal (treated with omeprazole plus nitrite), 2 animals in Group II (treated with omeprazole and omeprazole plus nitrite) and 4 animals in Group III (treated with water, nitrite, omeprazole and omeprazole plus nitrite). Therefore, positive points of this study are that malignant tumors were registered in the animals operated and submitted to duodenogastric reflux. And, in addition, tumors have also been reported in animals treated with nitrites, omeprazole and omeprazole plus nitrite.

In recent decades, most experimental studies have been carried out on mammals, due to their similarities with humans, in many ways. The conclusions of studies on animals, respecting ethical aspects, cannot be fully transposed to humans ^18^ ^,^ ^20^ . The results of this study again confirmed that the lesions are due to the presence of duodenogastric reflux. Therefore, the association of omeprazole did not offer any protective effect to the animals' gastric mucosa exposed to duodenogastric reflux.

## Conclusions

The occurrence of squamous hyperplasia, adenomatous hyperplasia and adenocarcinoma increased in frequency in Groups II and III, precisely in animals that underwent gastrojejunal anastomoses, which cause duodenogastric reflux. The association of omeprazole did not protect the development of proliferative lesions and cancer of the gastric mucosa induced by duodenogastric reflux in rats.
